# Supplementary material for: Cryo-EM reveals structural basis for human AIM/CD5L recognition of polymeric immunoglobulin M
Source: Nat Commun. 2024 Oct 30;15:9387. doi: 10.1038/s41467-024-53615-5 (PMC11525585; doi:10.1038/s41467-024-53615-5)
Supplement: Supplementary file 1 — Supplementary Information [file 41467_2024_53615_MOESM1_ESM.pdf]

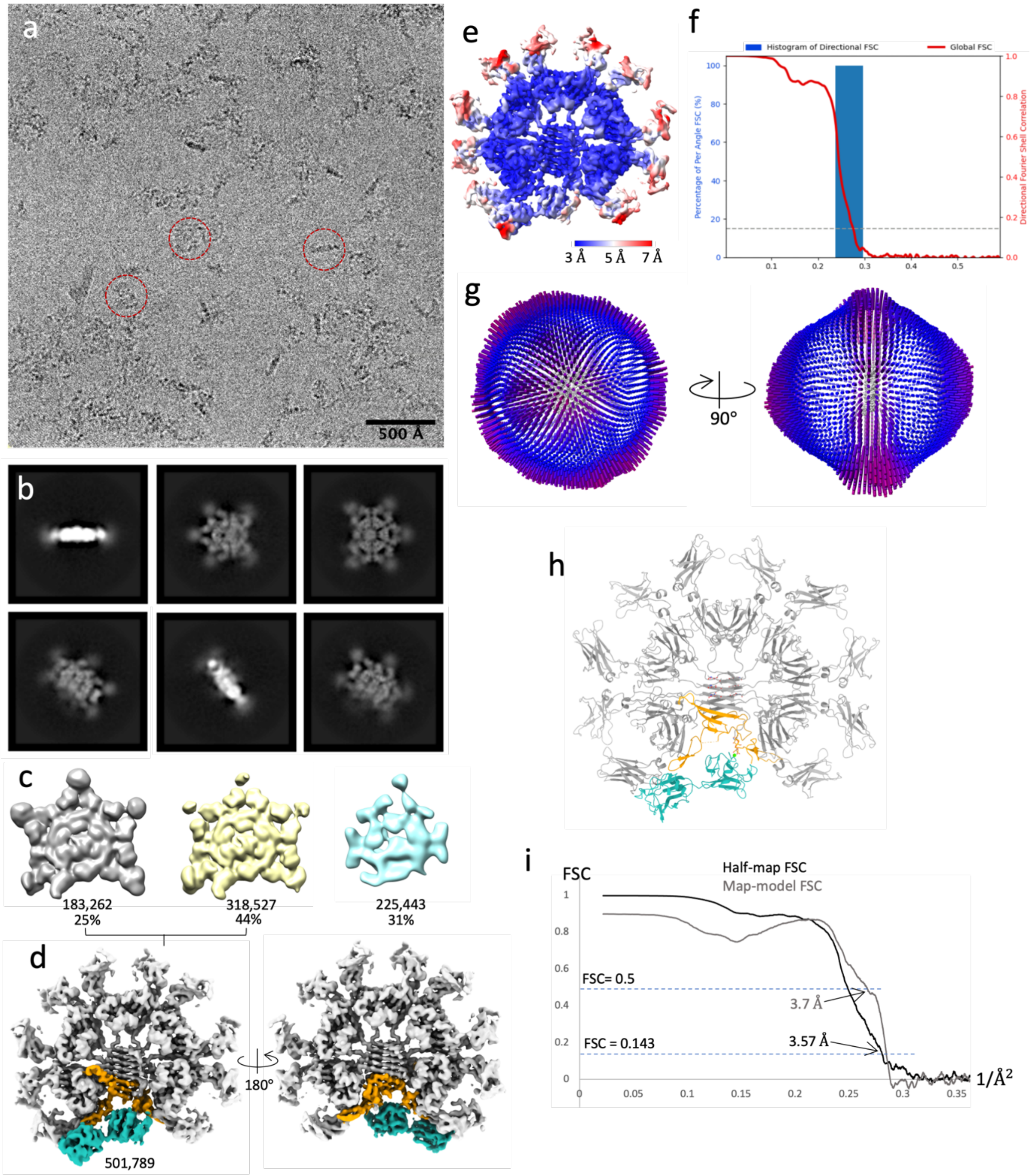

Supplementary Fig. 1. Single particle analysis of Fcμ/J/AIM complex. (a) A typical cryo-EM micrograph of the Fcμ/J/AIM complex with particles circled. (b) Typical 2D averages of the complex showing various orientations of the particles. (c) 3D classes of the complex. The first two were selected for further refinement in (d). Fcμ is coloured in grey, J chain in orange, and AIM in cyan. (e) Local resolution of the refined map calculated in Cryosparc. (f) 3DFSC histogram calculated in Cryosparc of the refined map showing overall isotropic resolution. (g) Angular distribution of the particles for the refined map. The left panel has the same orientation as (e). (h) Model of Fcμ/J/AIM complex. Fcμ chains are in grey, J chain is in orange and AIM in cyan. (i) Fourier shell correlation (FSC) with 3.57 Å resolution at 0.143 cut-off and map-model FSC showing 3.7 Å resolution at 0.5 cut-off for the model (calculated using Phenix program suite).

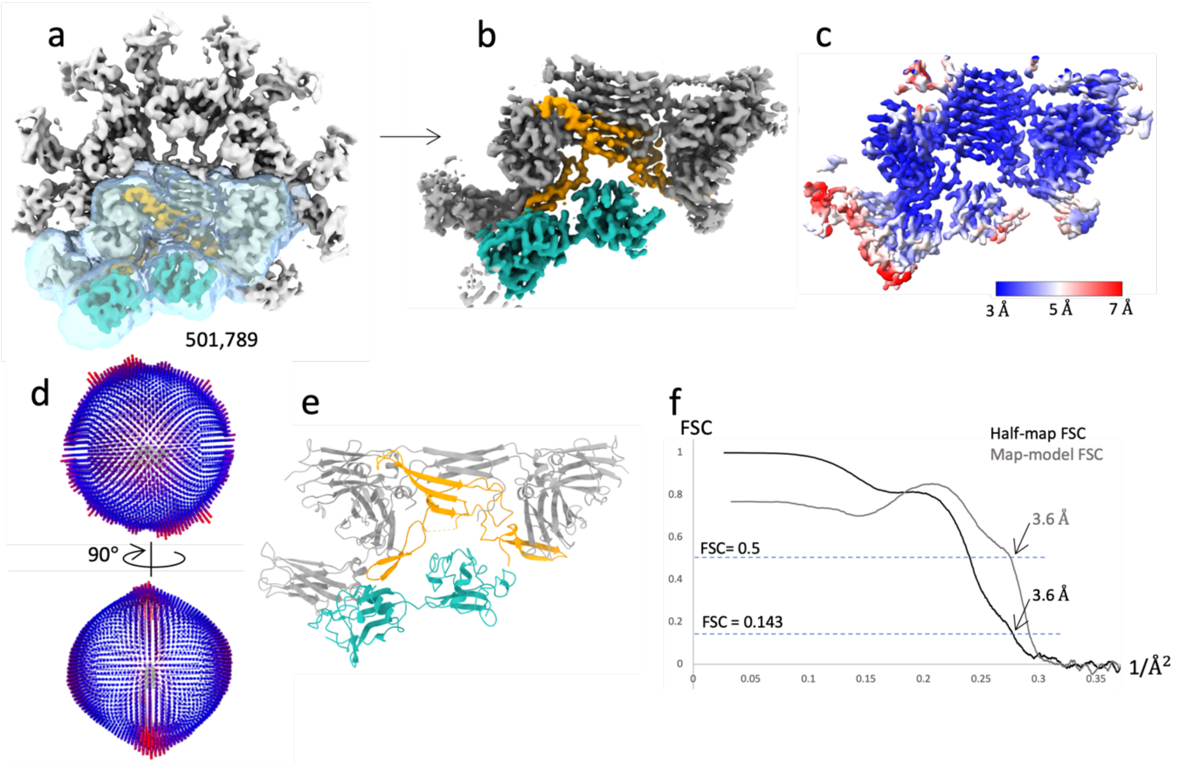

Supplementary Fig. 2. Single particle focused refinement at the IgM/AIM interface. (a) Refined complex map as in Supplementary Fig.1d showing the mask around the IgM/AIM interface applied to local refinement. (b) Postprocessed local refinement map. (c) Local resolution of the focused refined map calculated in Cryosparc. (d) Angular distribution of the particles for the focused refined map. The left panel has the same orientation as (b). (e) Atomic model for the focused refined map. (f) FSC plots with 3.6 Å resolution at 0.143 cut-off and map-model FSC plot showing 3.6 Å resolution at 0.5 cut-off (calculated using Phenix program suite).

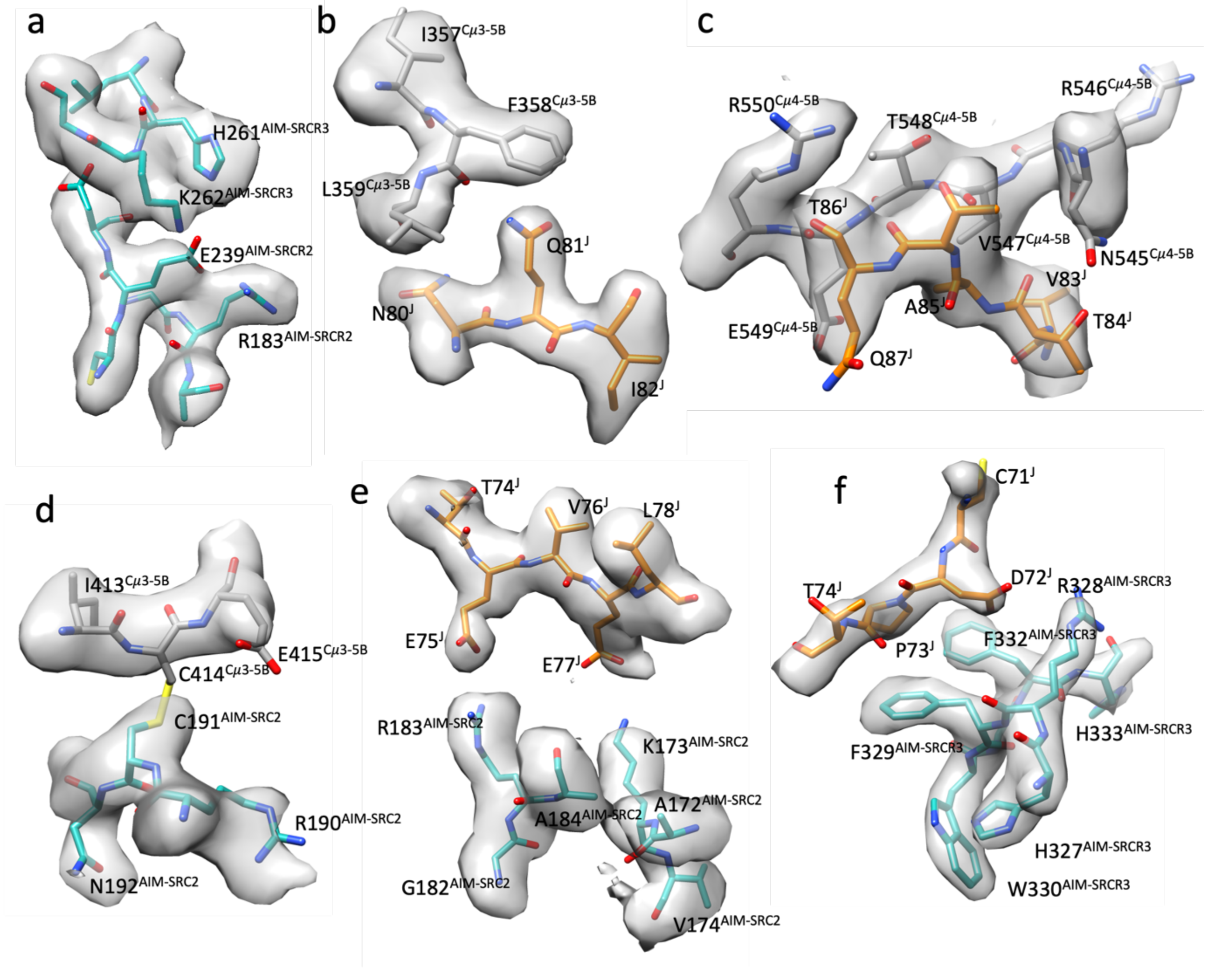

Supplementary Fig. 3. Density maps of key regions at Fcμ/J/AIM complex binding interface. (a) SRCR2-linker-SRCR3 interacting residues. Contour level t=0.2 (13σ). (b-c) Interacting residues at Fcμ/J interface. t=0.22 (14.5σ). (d) Interacting residues at Fcμ/AIM interface. t=0.2 (13σ). (e-f) Interacting residues at J/AIM interface. t=0.2 (13σ) in (e), t=0.27 (17.8σ) in (f).

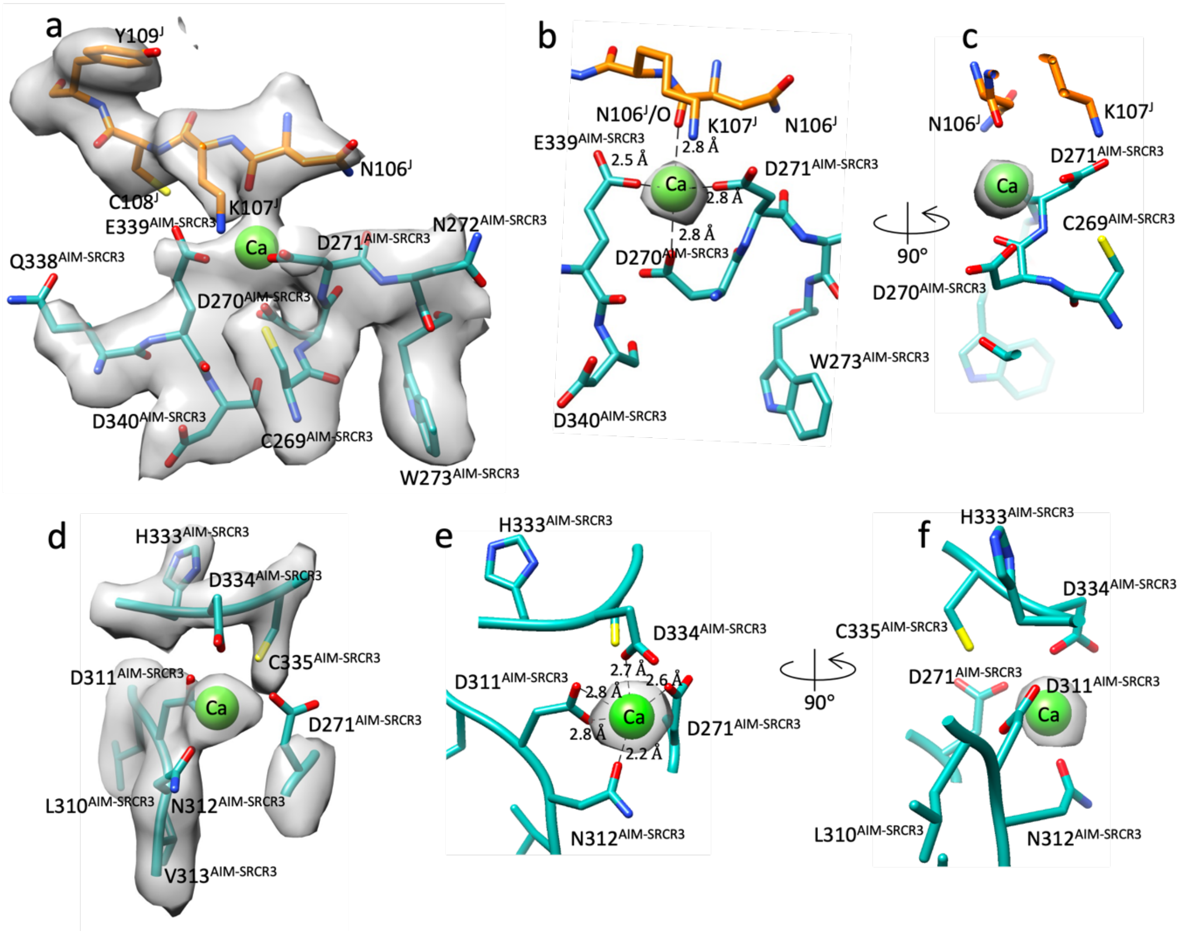

Supplementary Fig. 4. Local density maps for the two AIM SRCR3 calcium binding sites. (a-b) First calcium binding site that interacts with J chain. t=0.18 (11.9σ). (b) Same interactions in (a) with calcium-oxygen distances. The density of calcium ion is shown at a higher contour level (t=0.24 (15.8σ)) than (a). (c) Orthogonal view of (b). (d) Second calcium binding site. t=0.22 (14.5σ). (e) Same interactions in (d) with calcium-oxygen distances. The density of calcium ion is shown at a higher contour level (t=0.24 (15.8σ)) than (d). (f) Orthogonal view of (e).

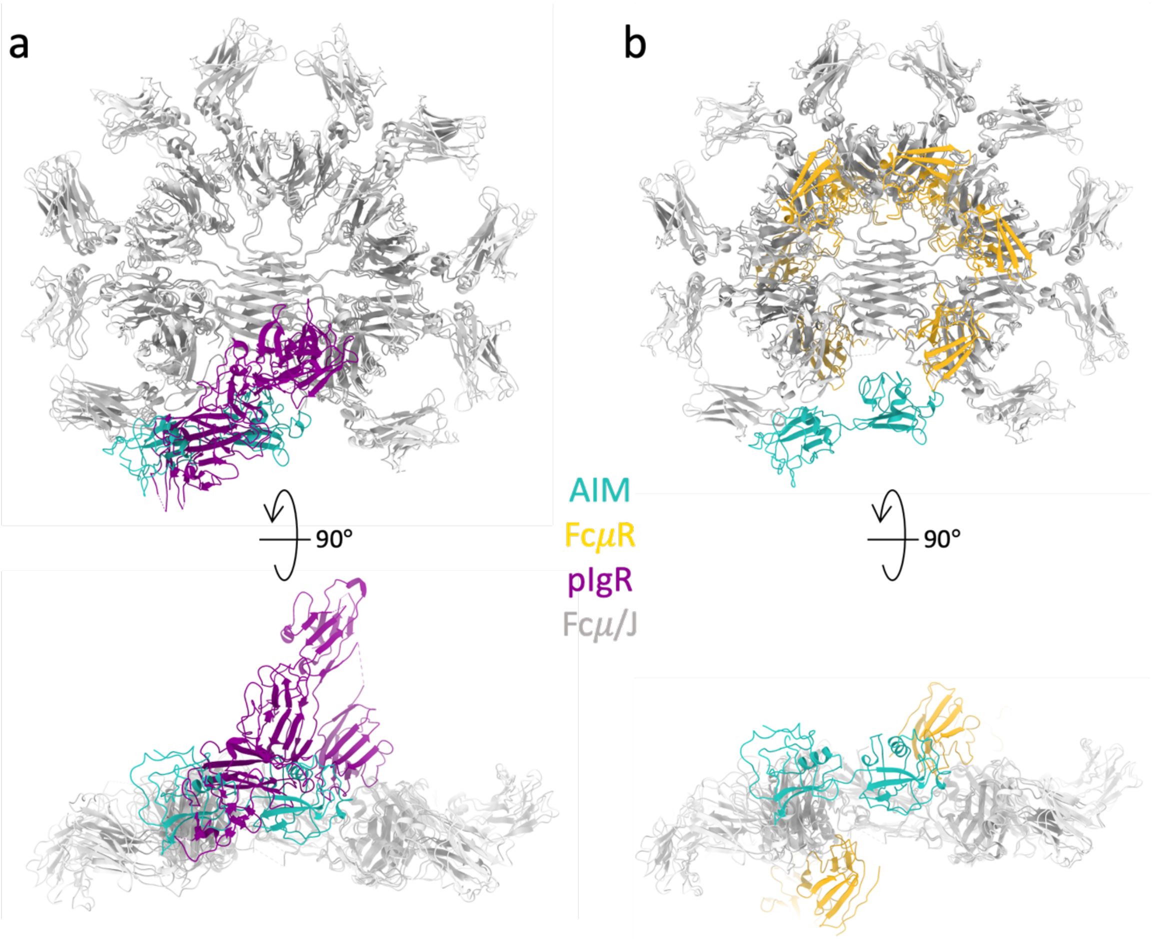

Supplementary Fig. 5. Compatibility of AIM and IgM receptors on IgM. (a) AIM and pIgR models at their respective IgM binding sites show significant clashes. (b) AIM and FcμR models at their respective IgM binding sites superposed on the same IgM core model without clashes.

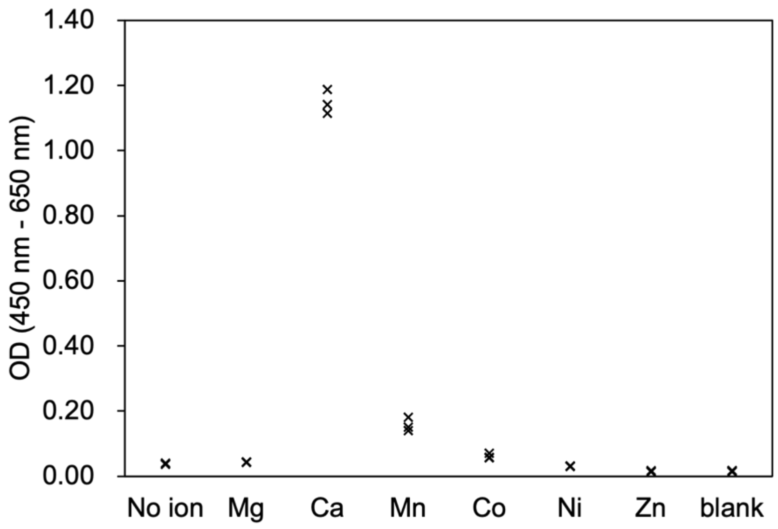

Supplementary Fig. 6. Effect of divalent cations on AIM/IgM binding in an ELISA-based assay. Three technical replicates for each cation are shown as individual data points. The data is representative of two biological replicates. Source data are provided as a Source Data file.

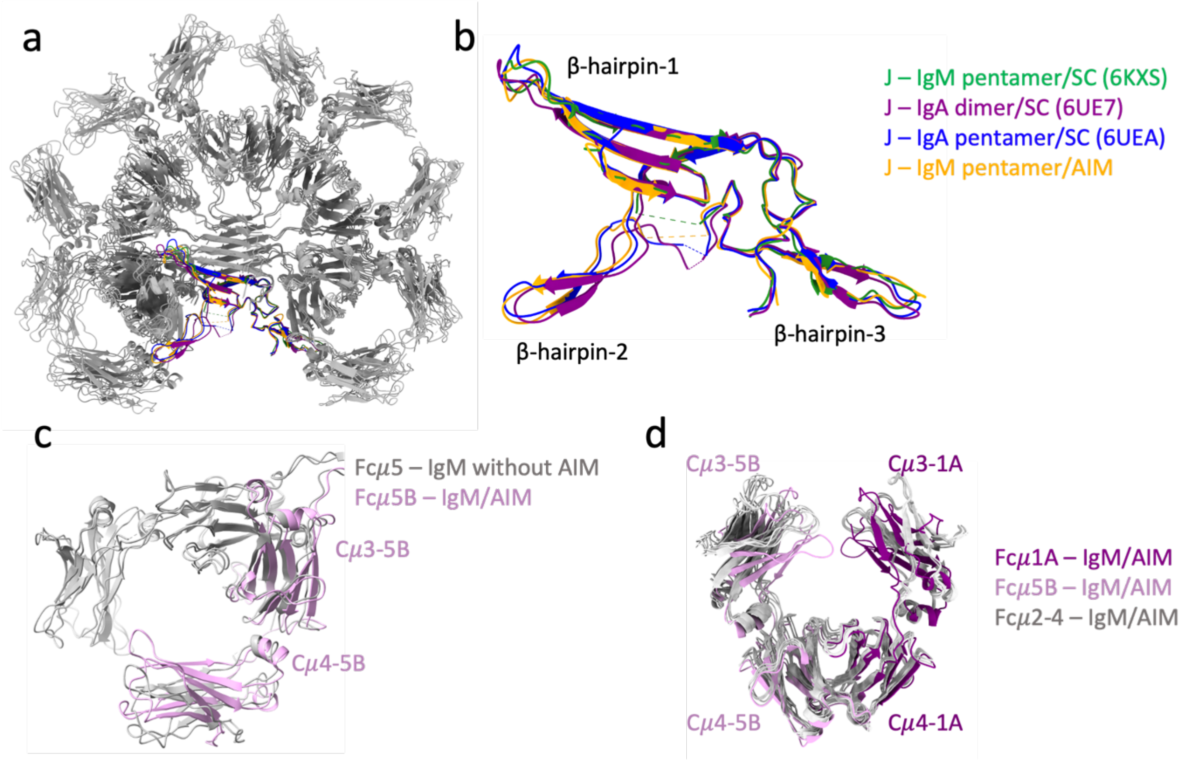

Supplementary Fig. 7. Comparison of J chain in Fcµ/J/AIM to other polymeric immunoglobulin structures. (a) Overlay of polymeric Ig structures aligned at J chains. The Fc regions are all in grey, J chains are in various colours listed in the legend in panel b. (b) Zoomed-in view of J chains in different structures. (c) Superposition of subunit Fcµ5 of IgM without AIM (in grey, pdb id 8ADY) and with AIM (Fcµ5A in grey, Fcµ5B in purple). (d) Superposition of the five Fcµ subunits of Fcµ/J/AIM complex. Fcµ1A in dark purple, Fcµ5B in light purple, the other Cµ3 and Cµ4 domains are in grey.

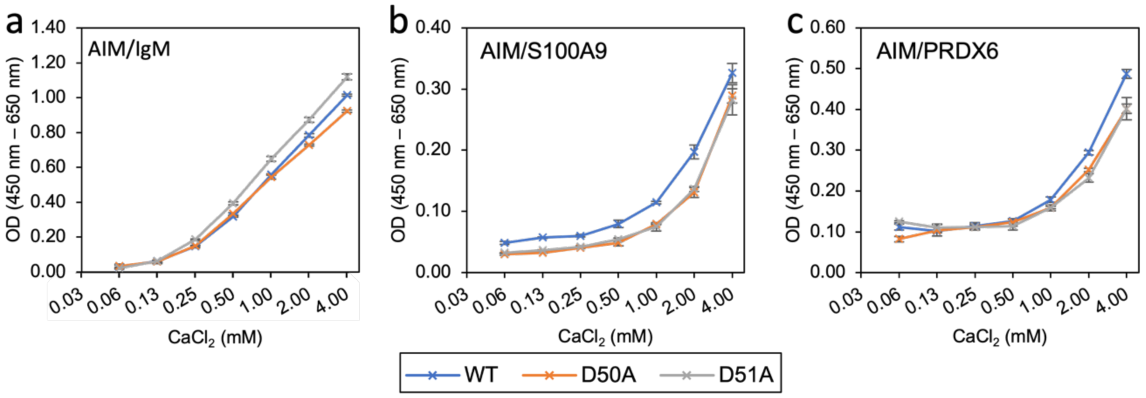

Supplementary Fig. 8. AIM binding to IgM and DAMPs is not affected by mutation of potential calcium-binding residues in SRCR1. (a) Binding between AIM (WT and mutants) and IgM at different calcium ion concentrations. (b) Binding between AIM (WT and mutants) and S100A9 at different calcium ion concentrations. (c) Binding between AIM (WT and mutants) and PRDX6 at different calcium ion concentrations. Error bars indicate the s.e.m. for three technical replicates. Data are representative of two biological replicates. Source data are provided as a Source Data file.

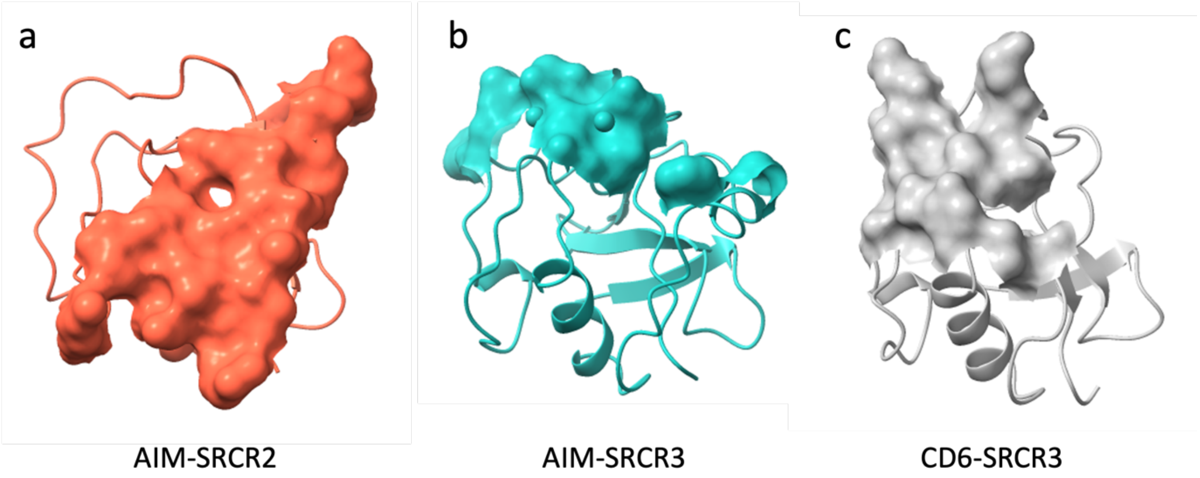

Supplementary Fig. 9. Ligand binding interfaces of three SRCR domains. (a) AIM-SRCR2 residues that contact IgM are shown as red surface. (b) AIM-SRCR3 residues that contact IgM are shown as cyan surface. (c) CD6-SRCR3 (pdb id 5A2E) with residues implicated in CD166 binding in grey (as described in Methods).

Supplementary Table 1. Cryo-EM data collection, processing and validation statistics

	global refinement Fcμ/J/AIM pdb id 8R83	local refinement Fcμ/J/AIM pdb id 8R84
**Data Acquisition**		
Voltage	300 kV	
Microscope	FEI Titan Krios	
Camera	K2, counting	
Calibrated magnification	59,101	
Electron exposure	52.4 e/Å^2	
Exposure rate	10.48 e/Å^2/s	
Number of frames per movie	40	
Energy filter slit width	20 eV	
Automation software	EPU	
Stage tilt	0°	
Defocus range	-1.2 to -3.5 μm	
Pixel size	0.846 Å	
**Data processing**		
Data processing packages	Relion, CryoSPARC, CrYOLO	
Initial particle images	3,266,779	
Symmetry imposed	C1	C1
Final particle images	501,789	501,789
Half-map FSC (0.5, masked/unmasked, Å)	4.08/4.42	4.20/8.08
Half-map FSC (0.143, masked/unmasked, Å)	3.57/4.13	3.60/4.32
Map B factor (Å^2)	134	98
Map sharpening method	Global B-factor sharpening in CryoSparc	
**Model Refinement**		
Initial model used	8BPF, AlphaFold prediction of human CD5L	
Refinement/validation packages	Phenix, Coot, CCPEM, Privateer	
Map-model FSC (0.5, masked, Å)	3.7	3.6
Map-model CC		
CC_mask	0.68	0.70
CC_volume	0.69	0.71
CC_peaks	0.56	0.51
CC_box	0.61	0.61
Model composition		
Non-hydrogen atoms	20415	7470
Protein residues	2608	951
Ligands	NAG: 8<br>CA: 2	NAG: 2<br>CA: 2
Validation		
MolProbity score	1.47	1.56
Clashscore	4.37	4.29
Rotamer outliers (%)	0	0
Ramachandran outliers (%)	0	0
Cβ outliers (%)	0	0
Planarity outlier	0	0
Chirality outlier	2	0
Bond length outlier	0	0
Bond angle outlier	4	2
